# Dendritic Cells/Natural Killer Cross-Talk: A Novel Target for Human Immunodeficiency Virus Type-1 Protease Inhibitors

**DOI:** 10.1371/journal.pone.0011052

**Published:** 2010-06-10

**Authors:** Maria Letizia Giardino Torchia, Elena Ciaglia, Anna Maria Masci, Laura Vitiello, Manuela Fogli, Andrea la Sala, Domenico Mavilio, Luigi Racioppi

**Affiliations:** 1 Department of Cellular and Molecular Biology and Pathology, University of Naples “Federico II”, Naples, Italy; 2 Laboratory of Molecular and Cellular Immunology, Department of Medical Science and Rehabilitation, IRCCS San Raffaele Pisana, Rome, Italy; 3 Section of Microbiology, Department of Experimental and Applied Medicine, University of Brescia, Brescia, Italy; 4 Laboratory of Clinical and Experimental Immunolgy, IRCCS, Istituto Clinico Humanitas, Rozzano, Milan, Italy; 5 Interdepartmental Center for Immunological Science (CISI), University of Naples “Federico II” Naples, Italy; New York University, United States of America

## Abstract

**Background:**

HIV-1 Protease Inhibitors, namely PIs, originally designed to inhibit HIV-1 aspartic protease, can modulate the immune response by mechanisms largely unknown, and independent from their activity on viral replication. Here, we analyzed the ability of PIs to interfere with differentiation program of monocytes toward dendritic cell (DCs) lineage, a key process in the inflammatory response.

**Methodology/Principal Findings:**

Monocytes from healthy donors were isolated and induced to differentiate *in vitro* in the presence or absence of saquinavir, ritonavir, nelfinavir, indinavir or amprenavir (sqv, rtv, nlfv, idv, apv, respectively). These drugs demonstrated a differential ability to sustain the generation of immature DCs (iDCs) with an altered phenotype, including low levels of CD1a, CD86, CD36 and CD209. DCs generated in the presence of rtv also failed to acquire the typical phenotype of mature DCs (mDCs), and secreted lower amounts of IL-12 and IL-15. Accordingly, these aberrant mDCs failed to support activation of autologous Natural Killer (NK) cells, and resulted highly susceptible to NK cell-mediated cytotoxicity.

**Conclusions/Significance:**

Our findings uncover novel functional properties of PIs within the DC-NK cell cross-talk, unveiling the heterogeneous ability of members of this class drugs to drive the generation of atypical monocyte-derived DCs (MDDCs) showing an aberrant phenotype, a failure to respond appropriately to bacterial endotoxin, a weak ability to prime autologous NK cells, and a high susceptibility to NK cell killing. These unexpected properties might contribute to limit inflammation and viral spreading in HIV-1 infected patients under PIs treatment, and open novel therapeutical perspectives for this class drugs as immunomodulators in autoimmunity and cancer.

## Introduction

HIV-1 Protease Inhibitors, that are included in the Highly Active Antiretroviral Therapy (HAART) of AIDS, has been specifically designed to inhibit HIV-1 aspartic protease [1], [2]. However, a number of reports have shown the ability of these drugs to modulate immune response by mechanisms largely independent from their activity on viral replication, a phenomenon that would account for part of the side effects observed in PIs-treated HIV-1-infected individuals[3], [4], [5], [6], [7], [8], [9], [10], [11], [12], [13].

The capability of PIs to exert immunosuppressive effects has been originally demonstrated in murine models of autoimmunity and infectious diseases [5], [10]. In the experimental autoimmune encephalitis, ritonavir treatment has been proved to decrease mononuclear cells infiltration, prevent the inflammatory response, and, in turn, ameliorate the clinical score [10]. Similar immunosuppressive effects have been reported in mouse infected by lymphocytic virus [5]. In this experimental model, ritonavir treatment limited the anti-viral T-cell cytotoxicity and prevented tissues damage induced by CD8+ T-cells. Several studies have also shown the ability of PIs to interfere with activation programs of human primary T-cells. Specifically, it has been demonstrated the effectiveness of rtv to inhibit the secretion of tumor necrosis factor alpha (TNF-α), and decrease the spontaneous and activation-induced susceptibility to apoptosis of uninfected peripheral mononuclear cells [4]. Pre-treatment with idv can also exert similar immunosuppressive effects on mononuclear cells isolated from peripheral blood of HIV-1+, or healthy individuals [13]. Although these studies provide unequivocal evidences for the immunosuppressive activity of certain PIs, the molecular events underlying these phenomena, as well as the cellular targets of PIs activity still remain not completely defined.

Dendritic cells (DCs) are a heterogeneous population of bone marrow-derived cells that orchestrate innate and adaptive immune responses [14], [15]. DCs are distributed in blood, peripheral tissues and lymphoid organs, and show the unique ability to activate and polarize naive T-cells. In peripheral tissues, DCs exist in two functional and phenotypic distinct states, immature and mature (iDCs and mDCs, respectively). iDCs are characterized by a high rate of endocytosis and low antigen-presenting capability. Activation of iDCs trigger their terminal differentiation program, namely maturation, which includes the switch from an antigen capture to a T-cell sensitizing mode, and the migration of activated cells, via-lymphatic vessels, to T-cells rich areas of regional draining lymphonodes. Although these mechanisms are crucial to prime the adaptive response, in HIV-1 infection it plays a controversial role: the ability of DC to binds HIV-1 enables them to carry virions to lymphoid tissues, and rapidly transmit them to nearby T-cells, in the form of an infectious synapse [16].

Beside their ability to prime naïve T-lymphocytes, DCs are also able to interact with autologous Natural Killer (NK) cells: the functional interaction between these two important cellular component of innate immunity, namely DC-NK cell cross-talk, is a key node of the cellular network regulating the links between innate and the adaptive immune response [17], [18]. Depending on DC/NK ratio, the final outcome of this interaction can vary from a progressive accumulation of activated DCs to their eradication from inflamed tissue [19]. The activation of DCs determines a progressive accumulation of a variety of cytokines, including IL-12 and IL-15, which, in turn, act as potent inducers of NK cell activation and cytotoxicity. Although activated NK cells produce IFN-γ and TNF-α, cytokines able to drive DC maturation process, the NK cell cytotoxicity against autologous iDCs prevails in the presence of a larger number of NK cells compared to that aberrant mature or immature DCs. This feedback mechanism limits the inflammatory reaction, and gate the traffic of activated DC to draining lymph nodes. Thus, the DC/NK cell cross talk can be identified as a critical node of cellular network regulating the inflammatory response in tissues, and triggering the adaptive response in secondary lymphoid organs [20], [21]. Since the functional consequences of this interaction would interfere with viral spreading to naïve T cells by gating the traffic of infected DC to lymph nodes, the functional interactions between NK cells and DCs might represent a novel target for immune-intervention in HIV-1 infection.

To verify the ability of PIs to target the DC/NK cell cross-talk, we analyzed the effects of a panel of PIs on the activation programs of MDDC and on DC/NK cell interactions. Although all PIs tested in our study were able to affect the differentiation program of MDDCs, only sqv and rtv displayed remarkable effects on terminal differentiation of immature DCs. Noteworthy, DCs generated in the presence of rtv also resulted highly defective in the priming of autologous NK cells and displayed a high susceptibility to NK-mediated lysis.

## Materials and Methods

### Media and Reagents

The regular medium used throughout was RPMI 1640 (Invitrogen, San Diego, CA, USA) supplemented with 2 mM L-glutamine, 50 ng/ml streptomycin, 50 units/ml penicillin, and 10% heat-inactivated fetal calf serum (Hyclone Laboratories, Logan, UT, USA). Granulocytes monocytes-colony stimulating factor (GM-CSF) was purchased from Schering-Plough (Kenilworth, NJ, USA). Interleukin-4 (IL-4) was obtained from ImmunoTools (Friesoythe, Germany). Sqv, rtv, nlfv, idv and apv were obtained through the NIH (Bethesda, MD, USA), AIDS Research and Reference Reagent Program. These drugs were dissolved in ethanol and lipopolysaccharide (LPS) contamination was excluded by *Limulus* assay (E-toxate; Sigma, Italy). As controls, cells were either left untreated or treated with a comparable concentration of vehicle (ethanol) without HIV-1 protease inhibitor (Fig. S1). Preliminary experiments were performed to define the optimal dose of PIs (Fig. S2). Based on these findings, on previous reports[9], [22], and on levels measured in sera of treated individuals[23], PIs were used at 20 µM. The human leukemic cell line, K562 were obtained from the American Type Culture Collection (ATCC, www.atcc.org; catalog# CCL 243). Peripheral blood mononuclear cells (PBMC) were obtained by leukapheresis performed in accordance with the clinical protocol approved by the Institutional Review Board (IRB) of the National Institute of Allergy and Infectious Diseases. Each patient signed a consent form that was approved by the above IRBs, in accordance with the Declaration of Helsinki.

### Generation of monocyte-derived dendritic cells and isolation of NK

PBMC from healthy donors were isolated over Ficoll-Hypaque gradients (lymphocyte separation medium; MP Biomedicals, Aurora, OH, USA). NK cells were negatively selected by using an antibodies cocktail against lineages specific markers and magnetic beads (StemCell Technologies Inc.,Vancouver, BC, Canada). According to cytometry, typical purified NK cells were 97% pure. To generate activated NK cells, freshly isolated NK were cultured *in vitro* with recombinant IL-2 (rIL-2; Roche) at 200 UI/m for 6 days. To generate iDCs, CD14^+^ monocytes were positively selected from PBMC by immunomagnetic procedure (Miltenyi Biotec, Calderara di Reno, Italy). Immature MDDC were then obtained by culturing CD14^+^ cells at 10^6^ cells/ml in RPMI 1640 (Invitrogen), 10% FCS (Fetal Calf Serum), 50 ng/ml GM-CSF and 1000 U/ml IL-4 in the presence or absence of PIs. After 6–8 d of culture, iDCs were extensively washed to remove PIs, and terminally differentiated by incubation with LPS at 1 µg/ml (Sigma-Aldrich, St. Louis, MO, USA) for 24 h. All cell culture was conducted at 37°C in humidified 5% CO_2_ atmosphere.

### Flow cytometry

Conjugated monoclonal antibodies against CD14, CD1a, CD86, CD80, CD83, CD40, HLA-DR, HLA-ABC, CD11c, CD36, CD54 were purchased from BD Biosciences (San Diego, CA, USA); antibody toward DC-SIGN were obtained from NIH AIDS research and reference reagent program. For intracellular cytokines detection, Brefeldin A (5 µg/ml; Sigma) was added to the culture medium. Cells were collected, fixed, and permeabilized according to manufacturer's instructions (Caltag Laboratories, Burlingame, CA, USA). Antibodies toward TNF-a, IL-12p40 and IL-10 were from BD Biosciences. NK phenotype was assessed by flow cytometry. Antibodies to TCR-α/β (IgG1), TCR-γ/δ (IgG1), CD19 (IgG1), CD14 and CD107a, were from Becton Dickinson. FITC anti -CD3/PE-Cy5, –anti-CD56 were from Beckman-Coulter-Immunotech, Marseille, France. Recombinant Human NKp30/Fc and NKG2D/Fc Chimeras were purchased from R&D Systems. The secondary staining was performer with the PE-conjugated rat anti-mouse immunoglobulin (BD Biosciences). Data were collected using a FACSCalibur flow cytometer (Becton Dickinson, USA), and analyzed by FlowJo software (Treestar, Palo Alto, CA, USA).

### NK-cell cytotoxicity

#### ^51^Chromium release assay

1×10^6^ target cells (K562 or autologous DC) were labeled with 1mCi of Na^51^CrO_4_ for 1 h at 37°C. Cells were then washed twice with complete medium and incubated with NK effector cells. After 4 h incubation at 37°C, supernatants were collected and presence of Na^51^CrO_4_ quantified by using Microbeta Trilux Scintillation counter (PerkinElmer, Waltham, Massachusetts, USA). Percentage of cytotoxicity was calculated using the formula [experimental-spons]/[maximum-spons] ×100, where experimental spons is Na^51^CrO_4_ released from targets, and maximum is the Na^51^CrO_4_ detected in targets exposed to 10% SDS (Sigma-Aldrich).

### CD107a degranulation assay

Activated NK cells were cultured at 37°C, 5% CO_2_ with or without K562 cells in the presence of PE-conjugated CD107a antibody. After 1 h, 2 mM monensin (Sigma Aldrich) was added to cultures. After 3 hrs, cells were collected, washed with PBS, stained and analyzed by flow cytometry.

### Cytokines secretion

IL-12p70, IL-15 and IFN-γ in supernatants were measured by ELISA, according to manufacture's specification (R&D Systems and Biosource International).

### Proliferation Assay

Freshly purified NK cells were cryopreserved until required as responders.Experiments were performed in triplicate in 96-well round plates with complete medium. To avoid the carrying over of PIs, iDCs were extensively washed before being cultured for additional 24 hours in the presence of LPS. Than, mDCs were collected, washed and co-cultured with autologous NK at a constant concentration of 2×10^5^ NK cells/well with autologous mDCs (stimulators) in serial dilutions (10–1.50×10^3^ cells/well). [^3^H]Thymidine (0.037 Mbq per well; PerkinElmer) was added 18 hrs before harvest cell cultures, and incorporation of [3H]thymidine into the cells was quantified by Microbeta Trilux Scintillation counter (PerkinElmer).

### Statistical analyses

Statistical analyses were performed using ANOVA test, (StatView Software, Abacus Concepts, v1.03). Differences were considered significant when p<0,05.

## Results

### Phenotype of immature DC generated in the presence of HIV-1 protease inhibitors

**Figure 1:**
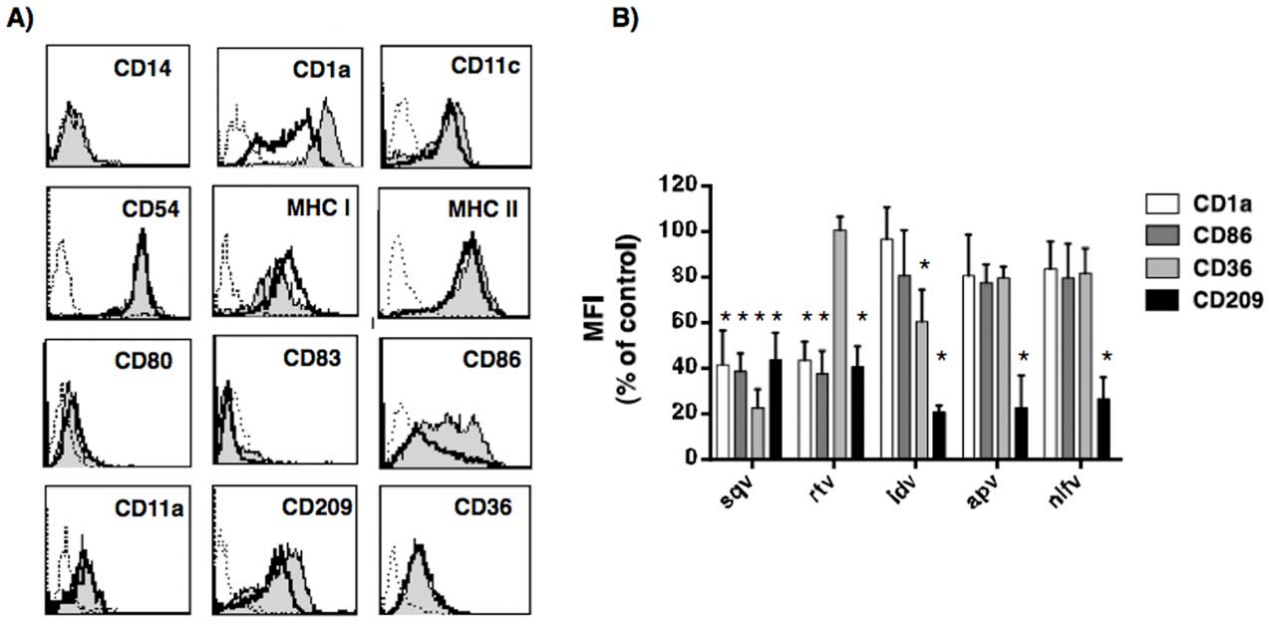
Phenotype of immature DCs generated in the presence of HIV-1 protease inhibitors. Purified CD14+ monocytes where cultured with regular medium containing GM-CSF (50 ng/ml) and IL-4 (1000 U/ml), with or without 20 µM of the indicated protease inhibitor. After 7 days, cells were collected, stained with the reported antibodies, and analyzed by flow cytometry. (A) Representative example for histogram profiles of iDCs generated in the presence (gray histograms) or in the absence (open histograms) of rtv. Open dot histograms represent isotype matched immunoglobulin staining. (B) Bars graph reports mean and standard deviation of the Mean Fluorescence Unit (MFU). Results are reported as the percentage of the MFU expressed on iDCs generated in regular medium, and are representative of ten independent experiments. (* p<0.005).

To investigate the ability of PIs to interfere with the differentiation program of monocytes toward DC lineage, we added sqv, rtv, idv, apv or nlfv to monocytes differentiation medium. After 7 days, the expression of differentiation makers (CD14, CD1a, CD11c, CD83), adhesion molecules (CD54, CD11a, CD11c), costimulatory molecules (CD80, CD86) and scavenger receptors (CD209, CD36) was tested by flow cytometry. Monocyte differentiation program toward DCs lineage includes down-regulation of CD14, and *de novo* synthesis of CD1a.These major changes were unaffected by the presence in the culture medium of idv, apv or nlfv. On the contrary, sqv, and rtv led to generation of iDCs with an atypical phenotype, including low levels of CD1a and CD86 (Fig. 1 and S1). The expression of CD36 was not affected by PIs with exception of sqv and idv, whose presence in the differentiation medium led to generation of DCs expressing significantly lower levels of CD36 (fig. 1). All PIs tested were unable to affect the expression level of CD54, CD11a, CD11c, MHC-I and -II, and CD80. On the contrary, all of them demonstrated the ability to affect the expression of CD209 (namely, DC-SIGN), a molecule involved in the binding, and spreading of the HIV-1 virions to T-lymphocytes [24] (fig. 1). All together these findings reveal a differential ability of PIs to interfere with differentiation program of monocytes and to drive the generation of iDCs with remarkable alterations in their phenotype.

### Responsiveness of immature DCs to bacterial endotoxin

**Figure 2:**
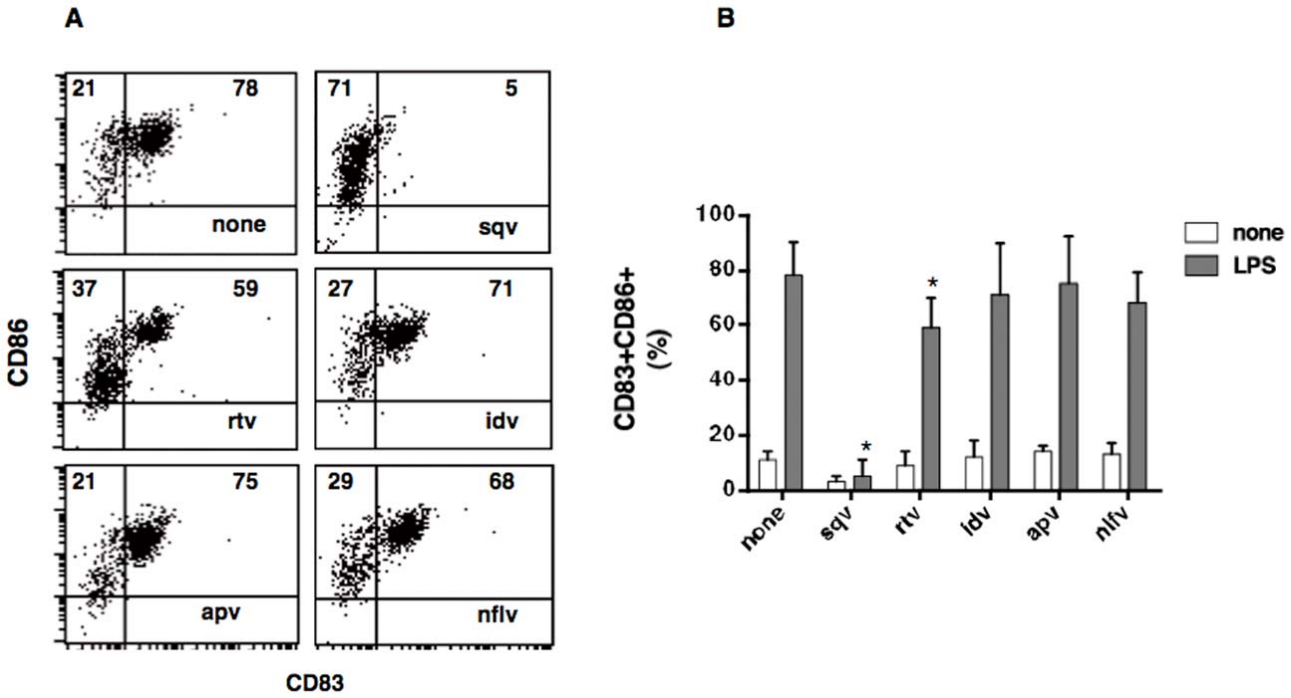
Responsiveness to LPS of immature DCs generated in the presence of HIV-protease inhibitors: immunophenotype. iDCs generated in the presence or in the absence of 20 µM of the indicated protease inhibitor were exposed to LPS (100 ng/ml). After 24 hours, DC were collected, stained with CD83 and CD86 antibodies, and analyzed by flow cytometry. (A) Representative example for dot plots showing CD83 and CD86 expression. (B) Bars graph report mean and standard deviation of CD83+CD86+ DC percentage calculated on ten independent experiments. (* p<0.005).

**Figure 3:**
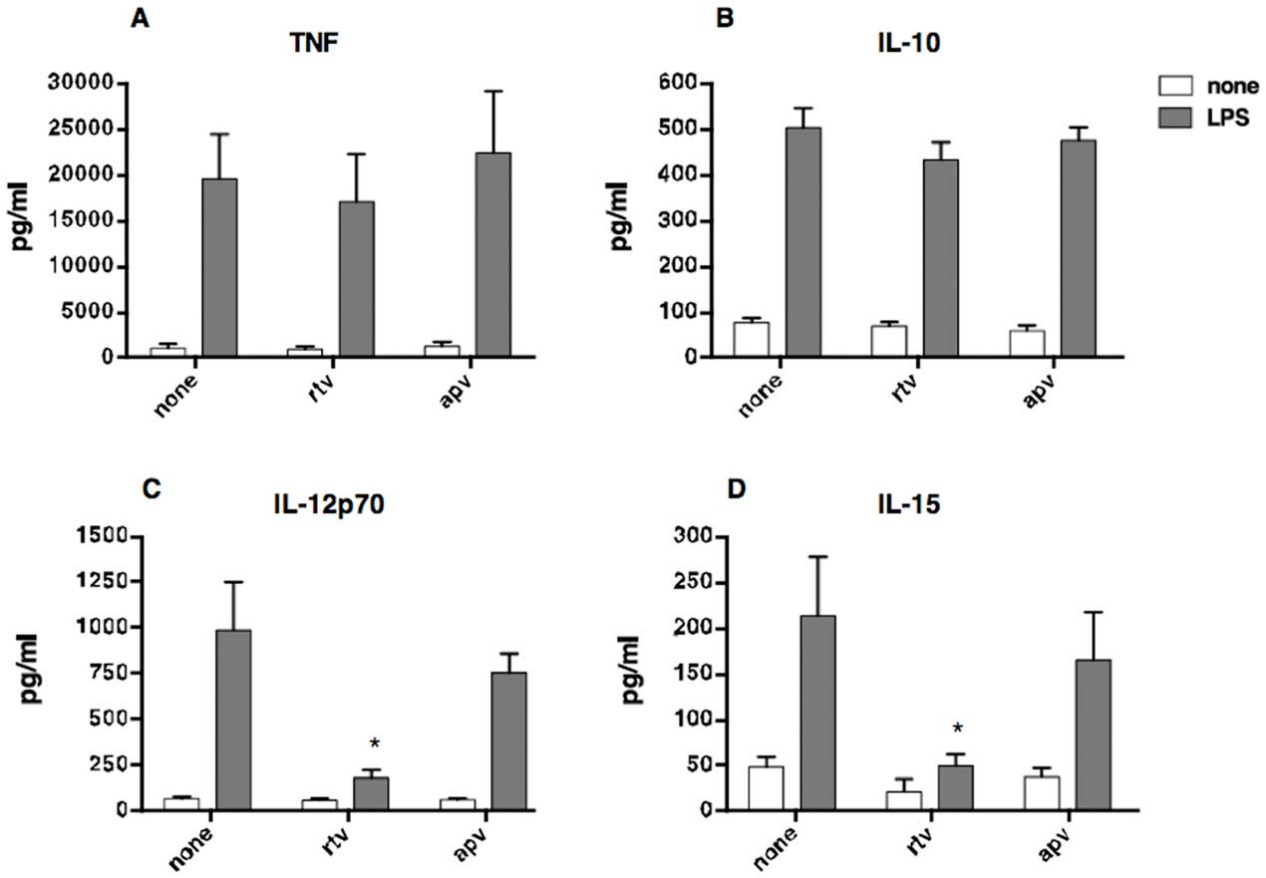
Responsiveness to LPS of immature DCs generated in the presence of HIV-1 protease inhibitors: cytokines release. iDCs generated in the absence (none) or presence of ritonavir (rtv) or amprenavir (apv) were collected, washed and exposed to LPS (100 ng/ml). After 24 hours, cytokines were measured in supernatants by ELISA (A–D). Bars graph reports mean and standard deviation calculate on ten independent experiments. (* p<0.005).

To investigate the capability of DCs to secrete cytokines and terminally differentiate in their mature form, we exposed cells generated in the presence or absence of PIs to the bacterial lipopolysaccharide (LPS). After 24 hours, cells were collected and analyzed by flow cytometry. In addition, we measured cytokines accumulating in supernatants by ELISA. iDCs generated in the presence of idv, apv, nlfv or regular medium displayed a comparable *de novo* expression CD83 and up-regulation of CD86, a typical change associated with the terminal differentiation process [15](fig. 2). On the contrary, iDCs-sqv and iDCs-rtv failed to up-regulate these molecules, and thus to fully differentiate in response to LPS (fig. 2). We decided to focus our studies on rtv rather than on sqv, considering that the first one is the elective choice in anti-retroviral therapy (ART)[1]. Moreover, to validate the specificity of rtv-dependent effects, we used apv as control based on the fact that this drug did not affect DC differentiation and bacterial endotoxin response. To further investigate the ability of DCs to undergo terminal differentiation upon stimulation with LPS, we measured the amount of pro and anti-inflammatory cytokines in supernatants. The results documented the comparable ability of DC-rtv, DC-apv and DCs generated in regular medium to secrete TNF-α and IL-10 in response to an optimal dose of LPS (fig. 3A–B). On the contrary, the presence of rtv, but not apv, in the differentiation medium led to generation of DC that secreted remarkable lower amounts of IL-12p70 and IL-15 (fig. 3C–D).

### DC-rtv failed to prime NK cell

**Figure 4:**
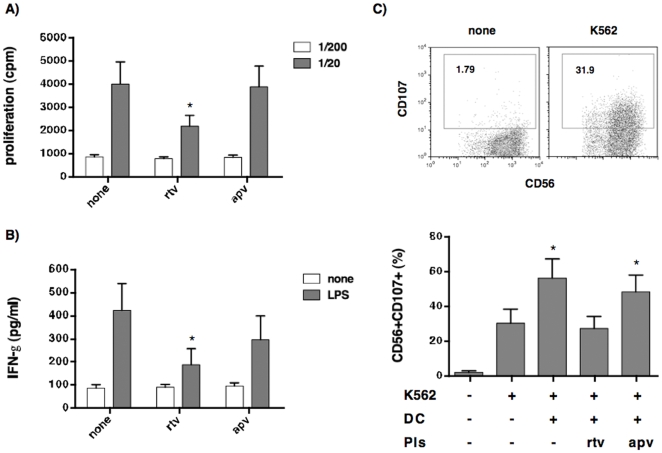
DCs generated in the presence of ritonavir fail to activate autologous NK cells. iDCs generated in regular medium (none) or in the presence of PIs (rtv or apv) were exposed to LPS (100 ng/ml). After 24 hours, cells were collected, washed and co-incubated with autologous NK. (A) Proliferative response of NK cells co-cultured for 4-days with autologous DCs in serial dilutions. Mean and SD are calculated on 5 independent experiments. (B) Accumulation of IFN-γ in supernatants derived from 24-hours NK/mDC co-cultures. Mean and SD are calculated on 5 independent experiments. (C) NK cells were co-cultured with autologous mDC for 24 hours, before being tested for their ability to de-granulate in response to K562 cell line. Upper panels: representative dot plots showing CD107 expression in CD56+ NK cells cultured with or without K562 (left and right panel, respectively). Lower panel: bars graph report the percentage of CD56+/CD107+ NK cells primed with autologous DCs generated in the presence of rtv, apv or regular differentiation medium for 24 hours, and co-cultured with K562 for 4 hours. Results shown are representative of five independent experiments. (* p<0.005).

Given the central role of IL-15 and IL12p70 in DC-dependent NK-cell activation [25], [26], [27], we aimed to investigate whether or not rtv-treated DCs preserved their ability to interplay with NK cells. We first examined the ability of LPS-treated mature DCs (mDCs) to proliferate and to induce the secretion of IFN-γ by autologous NK cells. The impairment of IL12p70 and IL-15 secretion in response to LPS stimulation paralleled the deficiency of mDC-rtv in triggering the proliferation of autologous NK cells (Figure 4). In contrast, DCs generated in the presence of apv or regular medium showed comparable ability to induce NK cell proliferation and secretion of IFN-γ (Figure 4A and B). In line with previous reports [28], we reasoned that the lack of mDC cognate activation of autologous NK cells would also affect their ability to prime NK cell cytotoxicity toward a non-cognate susceptible target. To test this hypothesis, we co-incubated mDCs and NK cells for 24 hours, and subsequently analyzed the cytolytic potential of DC-primed NK-cells against the classical K562 target. Pre-incubation of NK cells with DC generated in regular medium or in the presence of apv resulted in a similar NK killing of K562 cell line (fig. 4C). On the other hand, DC-rtv severely affected the NK cell ability to eliminate K562 cell line. These findings unveil the ability of certain PIs, including rtv, to impair the DC/NK cell cross-talk.

### Mature DC-rtv showed high susceptibility to NK cell lysis

Cross-talk between DCs and NK cells implies the ability of DCs to prime resting NK cells and license them for cytotoxic functions. Conversely, resting and activated NK cells can edit immune response by tuning quantity and quality on mDC moving from inflamed tissues to secondary lymphoid organs [18], a phenomenon that can play a critical role to HIV-1 spreading to T-cells [29]. Therefore, it is of great potential interest to evaluate the impact of PIs on the ability of DCs to become resistant to NK cell-mediated lysis. In this regard, NKp30, one of the three natural cytotoxicity receptors, has been shown to be primarily responsible for the NK cell–mediated killing of autologous iDCs [28].

**Figure 5:**
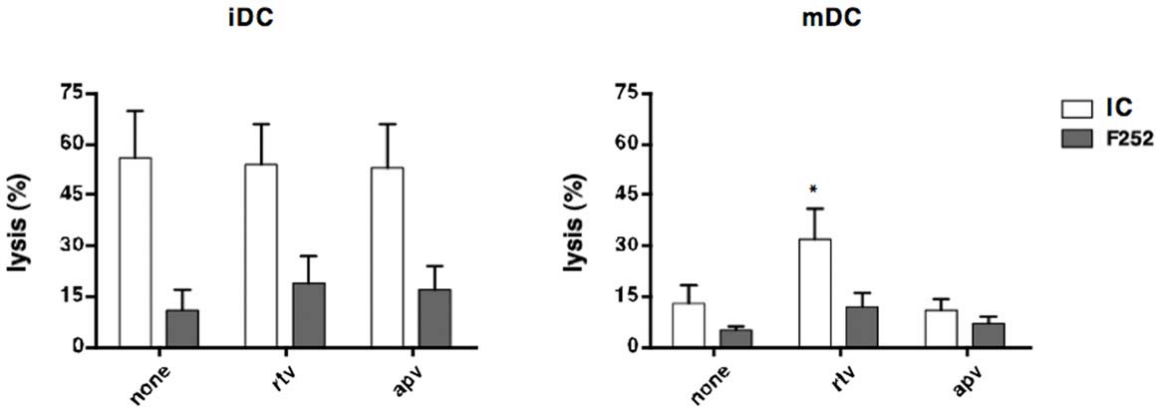
Mature DCs generated in the presence of ritonavir show high susceptibility to NK cell-mediated lysis. iDCs generated in the absence (none) or in the presence of PIs (rtv or apv) were exposed to LPS (100 ng/ml) for 24 hours to generate mDC. Then, aliquots of iDCs and mDCs were used as targets of NK for cytolytic activity in a Cr^51^-release assay. Open bars report the percentage of iDCs and mDC lysis by autologous NK cells (A and B, respectively). Grey bars show the percentage of lysis measured in the presence of saturating amounts of antibodies toward the activating NK receptors NKp30 or isotype control (F252 and IC, respectively). Mean and SD are calculated on five independent experiments. (* p<0.005).

Immature DCs generated in the presence of rtv, apv or regular medium displayed comparable susceptibility to NK-cell mediated lysis (figure 5A), a phenomenon that was reverted by masking NKp30, and in less extent, NK2GD (Fig. 5A). The terminal differentiation process induced by LPS resulted in a marked decrease of susceptibility to NK lysis (figure 5B). However, DCs generated in presence of rtv were still highly sensitive to NK lysis. Of note, masking of NKp30 protected mDC-rtv from lysis (figure 5B). Therefore, we reasoned that the atypical susceptibility of mDCs-rtv to NK lysis might be caused by an abnormal expression of NK activatory receptors on their surface. To test this hypothesis, we measured levels of activatory ligands expressed on the surface of mDCs by staining cells with NK2GD- and NKp30-Fc chimeric molecules. mDCs obtained from monocytes generated in the presence of rtv, apv or regular medium showed comparable staining for NK2DG-Fc. On the contrary, we found a higher expression of NKp30 ligands on mDCs generated in the presence of rtv, compared to those generated in regular medium or apv (Fig. S3). Thus, although unable to activate NK cells, DCs generated in the presence of rtv can be efficiently “edited” by NK cells through the NKp30 pathway, a mechanism that may limit viral transmission from infected DCs to unifected T cells in secondary lymphoid organs. Of note, even though the NK cell surface levels of NKp30 is markedly decreased in active and chronic phases of HIV-1 infection, the control of viremia by antiretroviral therapy (ART) lead to a normalization of this activating receptor on NK cells [29]. Therefore, a successful ART including rtv can not only suppress viral replication and restore the NKp30 expression on NK cells, but can also be important in the physiopathology of DC/NK cell cross-talk and in the control of viral spreading.

## Discussion

In this study, we examined the effects of PIs on monocytes differentiation toward DC lineage. We found that the presence of certain PIs inhibitors in the differentiation medium led to generation of iDCs showing an altered phenotype, including low levels of CD1a, CD86, CD36 and CD209. DCs generated in the presence of rtv also failed to acquire the typical phenotype of mDCs, and secrete lower amounts of IL-12 and IL-15 in response to LPS. Accordingly, they fail to support activation of autologous NK, and are highly susceptible to NK-mediated cytotoxicity.

Other authors have investigated the effects of PIs on DCs. Gruber et al. originally described the property of certain PIs to impinge phenotype and functionality of MDDCs [9]. These authors were unable to show any effect on the phenotype of iDCs generated in the presence of PIs. In addition, by testing the acute effects of PIs on iDCs, they found sqv, or rtv unable to interfere with up-regulation of CD86 induced by LPS. According with them, we confirm the inability of PIs to affect the expression levels of CD11c, CD86 and HLA-DR. However, thanks to an extensive immunophenotypical analysis, we demonstrate the differential ability of certain PIs to drive generation of iDCs expressing remarkable lower levels of CD1a (sqv and rtv), CD36 (sqv and idv), and CD209 (sqv, rtv, idv, nlfv and apv). All these markers were not tested in the previous report [9]. Furthemore, by testing the effects induced by prolonged exposure of differentiating monocytes to PIs, we reveal for the first time the property of certain member of this class drugs to limit the ability of iDC to up-regulate CD86 and CD83 in response to LPS treatment. The effect of acute exposure of iDCs to PIs have been also tested by Whelan et al., which documented the ability of idv to interfere with kinetics of CD83 expression [22]. Again, the different experimental conditions used in our study might account for the apparent discrepancy between our data and those reported by Whelan et al. Finally, these authors also demonstrated a decrease in CD209 expression on iDC exposed to idv[22]. We confirm and extend this early observations to other member of this class drugs, identifing CD209 expression as a common and relevant target of PIs action on DCs.

Monocytes differentiation toward iDCs is a plastic process that can be shaped by microenvironment, and led to generation of DCs varying in their phenotype and functional properties. Several lines of evidences indicate that pro-inflammatory ability of MDDCs correlates positively with the expression level of CD1a [30], [31], [32]. Our findings document for the first time the property of certain PIs to support generation of CD1a^low^ DCs, which secrete normal levels of TNF-α, and lower amounts of IL-12p70 and IL-15. Similarly to that recently observed in MDDCs differentiating in hypoxic conditions [31], our data suggest that certain PIs might uncouple the ability of DC to promote local inflammation, from their capability to fully differentiate, and drive the Th1-polarized adaptive response. The ability of these drugs to prevent the generation of CD1a^high^ MDDCs might be beneficial in chronic HIV-1 disease to limit the inflammatory response, and ameliorate the severe loss of lymphocytes.

Chronic HIV-1 infection is associated with an increased risk for metabolic disorders and cardiovascular diseases, including lipodistrophy, and atherosclerosis [33], [34]. PIs treatment has been originally proposed to cause this phenomenon [34]. Accordingly, the ability of certain PIs to up-regulate CD36 on human mononuclear cells, has been identified as the relevant molecular defect accounting for the disturbance of lipid homeostasis, and, in turn, for the development of atherosclerotic plaque [34], [35], [36]. Although the association between HIV-1 infection and the increased expression of CD36 has been more recently confirmed in a large cohort of HIV-1 infected patients, a careful statistical analysis cast doubts on the real impact of PIs treatment in the pathogenesis of this abnormality [34], [37]. In our experimental conditions, PIs were unable to induce detectable increase in the levels of CD36 expression. On the contrary, the presence of sqv, or idv in the differentiation medium led to generation of DCs expressing significantly lower amounts of CD36. These data corroborate previous observations from Serghides et al., which demonstrated the ability of PIs to affect CD36 expression on human myeloid cell lines. Most notably, these authors also show a decrease of CD36 expression on circulating monocytes of healthy, and HIV-1-infected individuals treated for seven days with an ART, including ritonavir, nelfinavir or lopinavir/ritonavir [38]. All together these data prompt us to hypothesize that members of PIs class drugs display a differential ability to impinge the homeostatic regulation of CD36 expression on different cell types. Thus, the net effect of their treatment on lipid metabolism has yet to be completely understood.

All PIs tested in our study show a common property to drive differentiation of monocytes toward generation of DCs expressing low levels of CD209, namely DC-SIGN. Due to the high affinity for the viral glycoprotein gp120, DC-SIGN can bind HIV-1 virions also when present in tissues at very low levels. The engagement of DC-SIGN triggers the phagocytosis of virus particles into intracellular non-lysosomal compartments characterized by low pH, the presence of the tetraspannis CD81 and CD9, and no MHCII. A fraction of these virions are retained in those compartments until they are ‘recycled’ to plasma membrane, where they are released in to the immunological synapse to infect permissive CD4+ T cells [16], [24], [39]. Thus, the ability of PIs to decrease the expression of DC-SIGN on cells surface of DC would enables them to intercept one of the most pernicious routes used by HIV-1 to infect antigen-specific T-cells.

An increasing number of reports identify DC-NK cell cross-talk as a relevant node of cellular network linking the innate with adaptive immune system [18]. However, several authors documented abnormalities in DC-NK cell interactions in HIV-1 infection, including defects in the processes of reciprocal activation [29], [40], [41]. We demonstrate that certain PIs can exert direct effects on DC-NK cell cross talk. Specifically, we found that DCs generated in the presence of rtv fail to secrete optimal amounts of IL-12 and IL-15. This functional asset would explain impairments in the DC-mediated activation of autologous NK cells. Similarly, functional defects in MDDCs, including a failure in the release of optimal amounts of IL-10 and IL-12, and inability to activate autologous NK cells, have been also reported in HIV-1-viremic individuals [41]. Based on these similarities, contribute of PIs treatment in determining functional abnormalities of DCs would not be ruled completely out.

Activated NK cells can kill iDCs or mDCs that fail to undergo a proper maturation, a phenomenon originally referred as NK cell-mediated “editing” of DC that has been proposed to play a major role to shape the quality and strength of the adaptive immune response [18]. Due to the ability of DC to bind and carry virions to lymphoid tissues, in HIV-1 infection, the editing of DCs by autologous NK cells would also limit the spreading of virus from migrating infected-DC to naïve T-cells trafficking in lymphoid tissues. Therefore, the ability of certain PIs to sustain the generation of mDC displaying higher susceptibility to autologous NK cytotoxicity, reveal a novel property of these drugs that would be beneficial to restore an effective DC-editing, and, in turn, limit viral spreading in HIV-1 infection.

## Supporting Information

Figure S1 The PIs vehicle does not alter DC differentiation and responsiveness to LPS. Purified CD14+ monocytes where cultured with regular medium containing GM-CSF (50 ng/ml) and IL-4 (1000 U/ml), with or without 0.02% v/v of ethanol. After 7 days, cells were collected, washed and exposed to 1 µg/ml of LPS. After 24 hours cells were stained for the indicated antibodies and analyzed by flow cytometry. The experiment shown is one representative of three independent experiments.
(3.99 MB TIF)

Figure S2 rtv affects DC differentiation in a dose-dependent manner. Purified CD14+ monocytes where cultured with regular medium containing GM-CSF (50 ng/ml) and IL-4 (1000 U/ml), with or without the indicated concentrations of rtv (A) or apv (B). After 7 days, cells were collected, stained for CD1a and analyzed by flow cytometry. The results are representative of three independent experiments.
(1.70 MB TIF)

Figure S3 Expression of NKp30 and NKG2D ligands on mDC generated from monocytes differentiated in the presence or absence of PIs. iDC generated in the presence or absence of 20 µM rtv or apv were extensively washed and cultured with 1 µg/ml LPS to induce terminal differentiation. After 24 hours, cells were collected and stained for NKp30 ligands (A) or NKG2D ligands (B). Bar graphs reports average and standard deviation of three independent experiments.
(4.83 MB TIF)
