# Supplementary figures and images for: Dendritic Cells/Natural Killer Cross-Talk: A Novel Target for Human Immunodeficiency Virus Type-1 Protease Inhibitors
Source: PLoS One. 2010 Jun 10;5(6):e11052. doi: 10.1371/journal.pone.0011052 (PMC2883582; doi:10.1371/journal.pone.0011052)

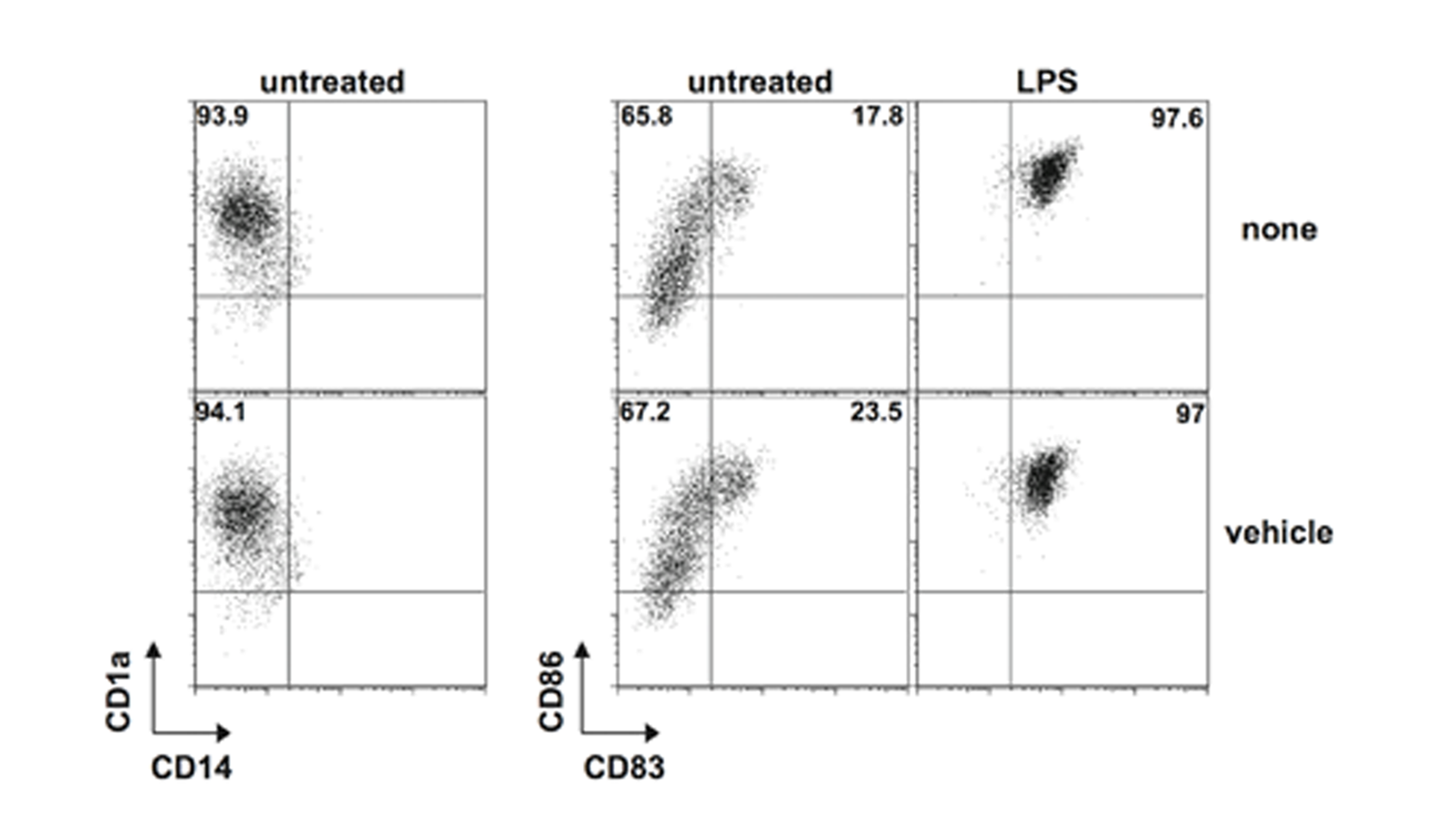

Supplement: Figure S1 — The PIs vehicle does not alter DC differentiation and responsiveness to LPS. Purified CD14+ monocytes where cultured with regular medium containing GM-CSF (50 ng/ml) and IL-4 (1000 U/ml), with or without 0.02% v/v of ethanol. After 7 days, cells were collected, washed and exposed to 1 µg/ml of LPS. After 24 hours cells were stained for the indicated antibodies and analyzed by flow cytometry. The experiment shown is one representative of three independent experiments. (3.99 MB TIF) [file pone.0011052.s001.tif]

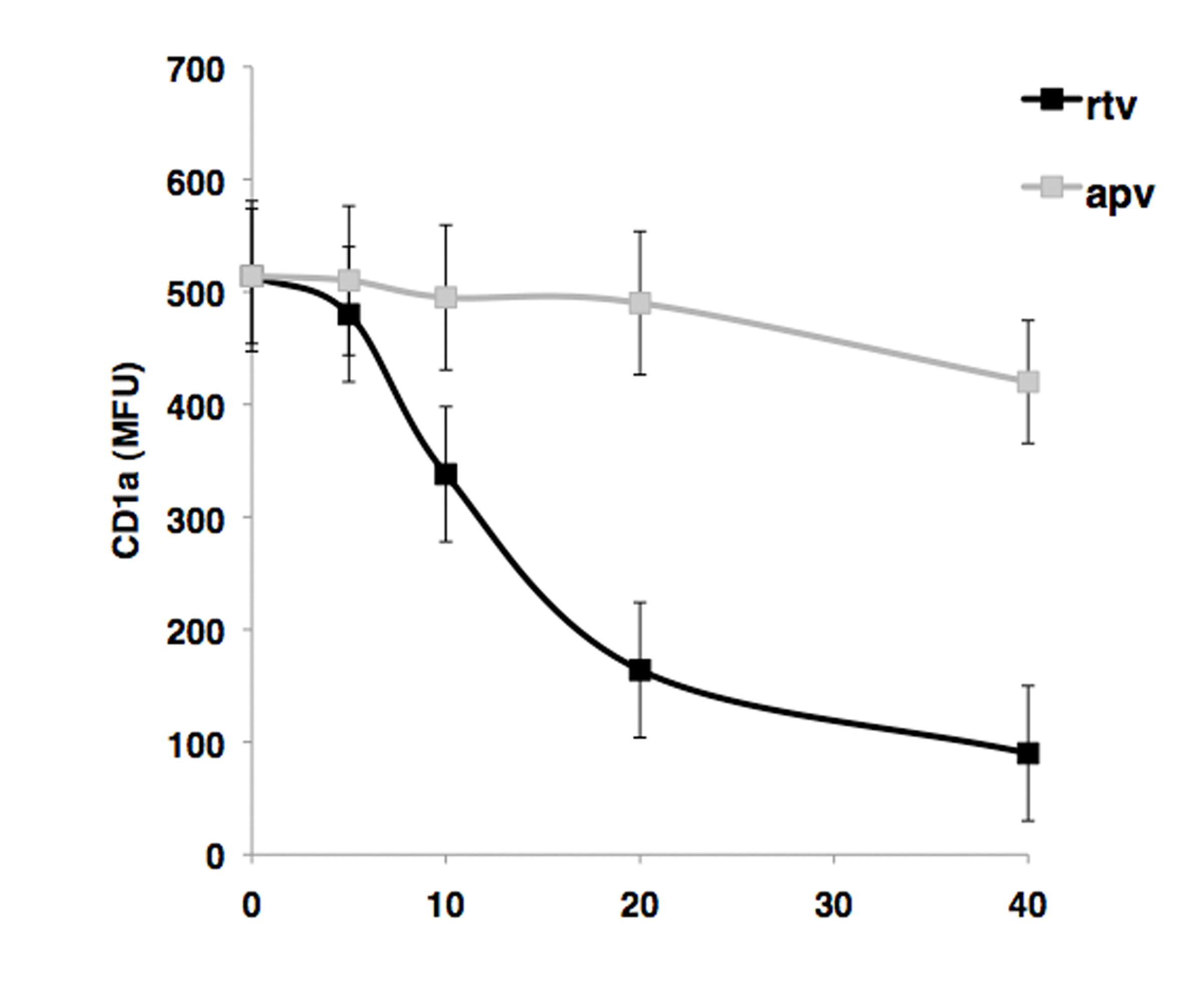

Supplement: Figure S2 — rtv affects DC differentiation in a dose-dependent manner. Purified CD14+ monocytes where cultured with regular medium containing GM-CSF (50 ng/ml) and IL-4 (1000 U/ml), with or without the indicated concentrations of rtv (A) or apv (B). After 7 days, cells were collected, stained for CD1a and analyzed by flow cytometry. The results are representative of three independent experiments. (1.70 MB TIF) [file pone.0011052.s002.tif]

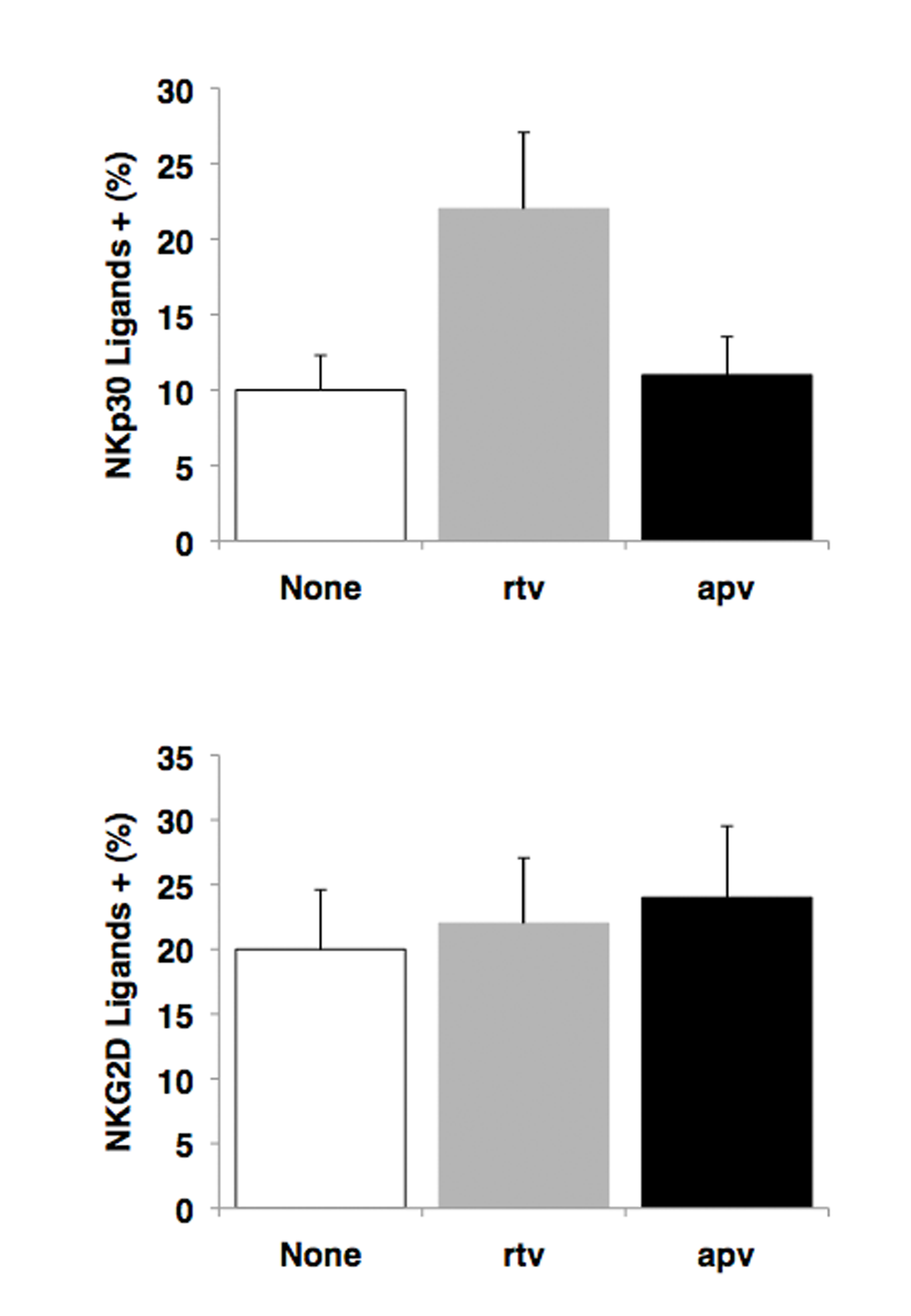

Supplement: Figure S3 — Expression of NKp30 and NKG2D ligands on mDC generated from monocytes differentiated in the presence or absence of PIs. iDC generated in the presence or absence of 20 µM rtv or apv were extensively washed and cultured with 1 µg/ml LPS to induce terminal differentiation. After 24 hours, cells were collected and stained for NKp30 ligands (A) or NKG2D ligands (B). Bar graphs reports average and standard deviation of three independent experiments. (4.83 MB TIF) [file pone.0011052.s003.tif]
